# Strategies towards digital and semi-automated curation in RegulonDB

**DOI:** 10.1093/database/bax012

**Published:** 2017-03-18

**Authors:** Fabio Rinaldi, Oscar Lithgow, Socorro Gama-Castro, Hilda Solano, Alejandra López-Fuentes, Luis José Muñiz Rascado, Cecilia Ishida-Gutiérrez, Carlos-Francisco Méndez-Cruz, Julio Collado-Vides

**Affiliations:** 1 Programa de Genómica Computacional, Centro de Ciencias Genómicas, Universidad Nacional Autónoma de México, A.P. 565-A, Cuernavaca, Morelos 62100, Mexico; 2 Swiss Institute of Bioinformatics and Institute of Computational Linguistics, University of Zurich, Andreasstrasse 15, Zurich 8050, Switzerland

## Abstract

Experimentally generated biological information needs to be organized and structured in order to become meaningful knowledge. However, the rate at which new information is being published makes manual curation increasingly unable to cope. Devising new curation strategies that leverage upon data mining and text analysis is, therefore, a promising avenue to help life science databases to cope with the deluge of novel information. In this article, we describe the integration of text mining technologies in the curation pipeline of the RegulonDB database, and discuss how the process can enhance the productivity of the curators.

Specifically, a named entity recognition approach is used to pre-annotate terms referring to a set of domain entities which are potentially relevant for the curation process. The annotated documents are presented to the curator, who, thanks to a custom-designed interface, can select sentences containing specific types of entities, thus restricting the amount of text that needs to be inspected. Additionally, a module capable of computing semantic similarity between sentences across the entire collection of articles to be curated is being integrated in the system. We tested the module using three sets of scientific articles and six domain experts. All these improvements are gradually enabling us to obtain a high throughput curation process with the same quality as manual curation.

## Introduction

Life science databases play a crucial role in the organization of scientific knowledge in the life science domain. The vastity and complexity of the life sciences require the presence of ‘knowledge brokers’ who identify, organize and structure information derived from experimental results and extracted from the literature. This important role is played by database curators, who act as intermediary between the producers of the knowledge (experimental scientist) and its consumers.

This important role requires highly-skilled individuals who have the biological expertise needed to recognize the crucial information that has to be inserted in a specific database. The complexity of the task, and the specific competences required, cannot be fully replaced by automated systems, if the aim is to obtain the same quality of results.

Nevertheless, it is becoming increasingly clear that this traditional approach cannot possibly cope with the deluge of new information being created by experimental scientists. PubMed, the reference repository of biomedical literature, at present contains >25 million bibliographical entries, and it grows at a rate of about two publications per minute. Even if individual databases do not need to process such a huge amount of literature, and can adopt partially automated triage strategies to quickly select only relevant articles, they are still left with a large amount of literature to process. Considering that for several databases only a very small subset of the information contained in an article is actually required for the curation process, it appears as a waste of time and resources that curators often have to read full articles in order to find those items that they need.

In this article, we propose automated strategies that help curators quickly locate that crucial information, and provide tools that support them in transposing this information from the article to the database. Through a combination of text mining and a user-friendly interface, based on text filters and partially pre-filled forms, curators can considerably enhance their efficiency, thus giving them the opportunities to process larger amount of documents, without losing the quality of the traditional manual curation approach. The ultimate goal is to obtain a high throughput curation with the same quality as manual curation, or, when such level of quality cannot be reached, at least be able to provide a quantifiable measure of the difference in quality.

The work presented here is part of an NIH-sponsored collaborative project (^‘^High Throughput Literature Curation of Genetic Regulation in Bacterial Models^’^) aimed at improving the curation process of the RegulonDB database. RegulonDB (http://regulondb.ccg.unam.mx/) is a database developed and maintained during years by our research group. This is the primary database on transcriptional regulation in *Escherichia coli* K-12 containing knowledge manually curated from original scientific publications, complemented with high throughput datasets and comprehensive computational predictions. Our team is also in charge of curating regulation of transcription initiation in EcoCyc (7), the *E. coli* model organism database with added curated knowledge on metabolism and transport (http://ecocyc.org/). One of the aims of the project is to integrate and further develop text mining technologies, previously developed as part of the OntoGene project (http://www.ontogene.org/), in order to improve the curation process of RegulonDB. OntoGene aims at improving biomedical text mining through the usage of advanced natural language processing techniques (19, 22).

In the context of the SASEBio project (Semi-Automated Semantic Enrichment of the Biomedical Literature), the OntoGene group previously developed a user-friendly interface (ODIN: OntoGene Document INspector) which presents the results of the text mining pipeline in an intuitive fashion, and allows a deeper interaction of the curator with the underlying text mining system (15).

In the rest of this article, we first briefly describe the purpose of the RegulonDB database (Section 2), then explain how our existing OntoGene relation mining system has been customized for RegulonDB (Section 3), including a brief overview of our ODIN curation interface (Section 4).

## Notes about RegulonDB

RegulonDB (6, 23) is a manually curated standard resource about the regulation of gene expression in *E.coli* K-12, the best characterized microbial organism. It aims at integrating in a single database all the scattered information about genetic regulation in this microorganism. RegulonDB has had a great impact on scientific research; >800 scientific publications with works related to analysis of gene regulation, bioinformatics, comparative genomics and systems biology have mentioned our published articles related to our database.

RegulonDB contains many elements about transcriptional regulation, such as promoters, transcription units (TUs), transcription factors (TFs), effectors that modify the behavior of TFs, active and inactive conformations of TFs, transcription factor binding sites (TFBSs), regulatory interactions (RIs) of TFs with their target genes/TUs, terminators, riboswitches, small RNAs and their target genes. It also contains regulatory molecules that do not bind to DNA but bind to RNA polymerase, such as the ppGpp and DksA, a small molecule and protein, respectively. Furthermore, we proposed new concepts such as ‘regulatory phrase’ which is the module in which multiple TFBSs are organized (23), and the ‘Genetic Sensory Response Units’ (4) that integrates gene regulation as a complete process starting with an environmental (or internal) signal, followed by the reactions of signal transduction, transcriptional regulation, and ending in a cellular response to the given signal. On the other hand, we have initiated the complex curation of data generated by massive expression experiments (high throughput), such as GSELEX and dRNA-seq experiments, among others.

On the basis of our previous curation activities for RegulonDB, we estimate that we could capture the information contained in much less than one fourth of all sentences available in the articles that we curated so far (6008 articles for RegulonDB 8.8). This estimate is based on the number of sentences behind our knowledge about TFs, TFBSs and their functions affecting genes and TUs. On the basis of this diagnosis, we decided to improve the efficiency of biocuration process by leveraging upon natural language processing technologies in text mining systems.

The curation process of RegulonDB starts with a triage phase, in which articles are identified in PubMed using specific keywords related to transcriptional regulation and operon organization in *E.coli K-12*. Additionally, the EcoCyc staff sends monthly suggestions of articles to curate. The abstracts of the articles are read and if selected, the complete articles are obtained. The traditional curation process involves reading each article and adding the corresponding data to EcoCyc (7) through their capture forms. The data are then shared with RegulonDB.

Biomedical text mining can be used to partially automate the process of biomedical literature curation by using sophisticated algorithms for discovering biomedical entities together with interaction and events in which they participate. A successful biomedical text mining system can be based on a pipeline which first discovers entities of interest in the text of a scientific article and subsequently looks for interactions between them. As described above, finding the unique database identifiers of the entities in focus is an important step in this process. Which database identifiers are used in this process depends largely on the application for which a text mining system is built, or in other words, the database for which the system is designed to extract information.

In order to accomplish the goal of digitally-assisted curation, we are working simultaneously on two main lines of research: (i) finding information relevant for curation, and present it in an adaptive interface, and (ii) use sentence-similarity techniques to create interlinks across articles thus allowing a process of knowledge discovery. These steps are described in detail in this article, together with the preliminary design of the integrated system.

## Text mining pipeline

The OntoGene group (http://www.ontogene.org/) at the University of Zurich (UZH) specializes in mining the scientific literature for evidence of interactions among entities of relevance for biomedical research (genes, proteins, drugs, diseases and chemicals). The quality of the text mining tools developed by the group is demonstrated by top-ranked results achieved at several community-organized text mining competitions (16, 19, 21).

In this section, we provide a brief description of the OntoGene system which is used to provide the basic text mining services required by the advanced applications described in this article. In particular OntoGene performs all of the standard text pre-processing tasks (identification of sections, sentence splitting, tokenization, part of speech tagging, lemmatization and stemming), and can optionally perform syntactic analysis using a dependency parser (which is then used for helping the recognition of interactions). The OntoGene pipeline has been described extensively in previous publications (14, 15, 17).

The OntoGene pipeline includes a module for entity recognition and disambiguation, based on an extensive database of biomedical terminology, which is used to identify mentions of domain entities and assign them an identifier from a reference database. We have separately designed a process to collect names of relevant domain entities from several life science databases and store them in an internal format which is used by the OntoGene pipeline to verify if any string in the document could be a reference to one of those entities (3). The OntoGene system takes automatically into account a number of possible minor variants of the terms (e.g. hyphen replaced by space), thus increasing the flexibility of term recognition. The annotation step automatically adds to the internal representation of the document a list of possible database identifiers for each term where a match was found (18).

Since it is possible (and quite frequent in this domain) that the same term indicates several possible entities, that is, corresponds to several different identifiers in a reference database, it is necessary to perform a step of disambiguation in order to (ideally) assign an unique identifier to each marked entity. A simple example of ambiguity is the name of a protein, which could also refer to the corresponding gene, but also could be the same for several different proteins which are orthologs across different species.

OntoGene uses a machine learning method to attempt the disambiguation of such ambiguous references. In order to train a machine learning system, reference annotations are needed, where the identifiers are unambiguously known. Typically, systems use a manually annotated corpus to learn to perform similar tasks effectively. However, annotated corpora are small and few, and might introduce a bias towards the particular choice of articles. OntoGene is based instead on a distant learning approach which takes life science databases as provider of the ‘ground truth’, which is used for learning a disambiguation approach (20). The basic assumption is that if the database provides a reference to entity A in article B, any term identified by the OntoGene pipeline as ‘A’ in the same article will be considered as correct. Even if this assumption might not be true in all cases, the number of incorrect cases is likely to be sufficiently small to be ignored by the machine learning algorithm, if a sufficiently large dataset is used for training.

**Figure 1.**
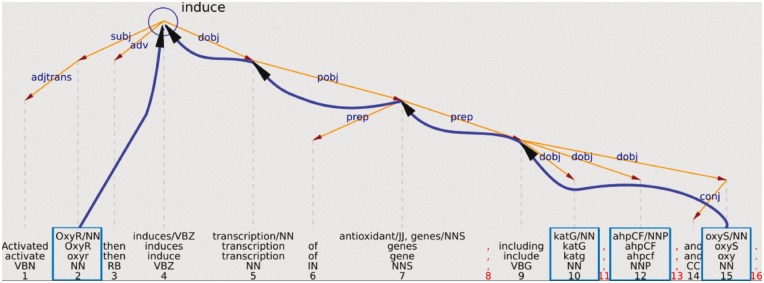
Example of using the syntactic structure to validate a potential relationship.

The OntoGene system performs a complete syntactic analysis of each sentence in the input documents. In most cases, it is relatively easy to recover from such analysis the information which is necessary to provide a relation type. For example, Figure 1 shows a simplified representation of the analysis of the sentence ‘Activated OxyR then induces transcription of antioxidant genes, including katG, ahpCF, and oxyS’. This sentence mentions interactions between a transcription factor (OxyR) and the genes katG, ahpCF, and oxyS. From the graphical representation it can be intuitively seen that the word which indicates the interaction verb ‘induce’ can be recovered as the uppermost node at the intersection of the syntactic paths leading to the arguments (only the interaction between OxyR and OxyS is explicitly indicated in the figure).

Another planned addition to the system is a module capable of computing semantic similarity between sentences across the entire collection of articles to be curated (semantic linking). Currently, when doing biocuration, the experts read one by one a set of topic-related articles to annotate relevant information. This technique works well in the sense that relevant information is identified but having to read the whole article sequentially is very time consuming. So, based on the fact that the documents have several topics in common, we propose to complement the current curation approach with a new approach based on cross-linked sentences on a collection of related articles. Therefore, we have designed a system that uses sentence similarity to link sentences about the same subject across all the articles in the set. For instance, complex sentences (like examples a, b and c) will be related, since they are about the same topic:

- *The oxidized form of OxyR is a transcriptional activator of a multitude of genes that assist in protecting the cell from oxidative damage(*26*).*
- *Activated OxyR then induces transcription of a set of antioxidant genes, including katG (hydroperoxidase I), ahpCF (alkylhydroperoxidase), dps (a non-specific DNA binding protein), gorA (glutathione reductase), grxA (glutaredoxin I)and oxyS (a regulatory RNA)(*27*).*
- *A hallmark of the E. coli response to hydrogen peroxide is the rapid and strong induction of a set of OxyR-regulated genes, including dps, katG, grxA, ahpCFand trxC(*28*).*

This way the regular reading is modified, allowing the reader to choose one sentence of interest and jump/navigate through other articles, guided by the current topic of interest.

**Figure 2.**
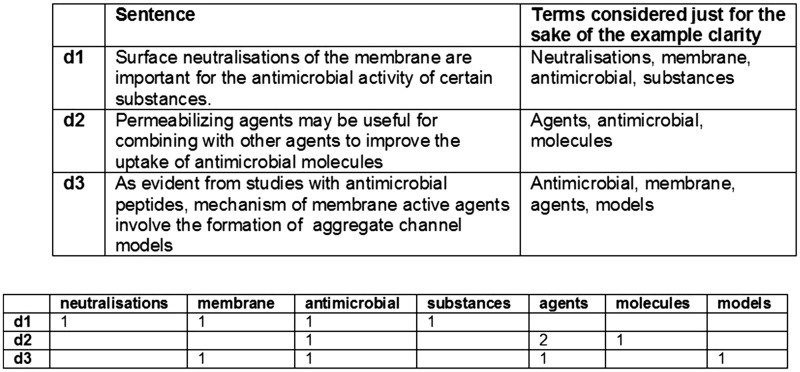
Simplified example of distributional vectors.

This first design of the similarity engine is based on the simplest distributional representation of the sentences. A sentence is characterized by the frequency of appearance of each word on it, and each of these counts represents a dimension in a vector that states for the sentence, resulting in a Vector Space Model (VSM). Once each sentence is transformed to a vector, their proximity can be obtained by computing the cosine (We are using Efficient Java Matrix Library (6) for the matrix computations.) between each two vectors (sentences) and this proximity in the Euclidean space should correspond with their proximity in their meaning based on the bag of words hypothesis. This hypothesis states that frequencies of words in a document tend to indicate the similarity of the document to another document (documents are phrases in this case) (11, 24). A simplified example can be seen in Figure 2.

This is a well-known strategy to measure text similarity and although state-of-the-art strategies are more sophisticated, differences are mainly in the way vectors are constructed. Therefore, our plan is to improve this particular component through new versions that try other approaches to build up these vectors. It is worth to note that, before transforming sentences into vectors, an important step is to apply a series of text ‘normalization’ tasks in order to alleviate the huge variability of natural language. Some of these tasks are intended to reduce words to their stem and in that way lower the space dimensionality by collapsing words with the same lexeme (snowball stemmer (7)), and to identify words of similar grammatical function by applying a Part-of-Speech tagging (POS-tagging) (using the Stanford POS tagger (19)).

We are interested in sentence similarity in the context of similarity of their meaning. In this sense, besides having a qualitative evaluation, it is desirable to have a quantitative evaluation of how well our system computes the similarity between two given sentences. That is why the new version of the similarity comparator module is being designed not just to incorporate more sophisticated state-of-the-art strategies for score sentence similarity, but also to be easily evaluated. This new version that we are currently working on includes a different approach to the measurement of semantic similarity. In the new version, instead of relying on a single metric, we are opting for a more robust strategy consisting in combining several similarity metrics, semantic and syntactic. At the time of this report, we have explored the use of a lexical database, Wordnet (10), which we use to compute the semantic similarity between a pair of words (one word for each sentence of evaluated pair). So far, the measure which has given us better results is Lin98 (9). We have also tested another semantic metric based on word embeddings, using the Glove library (13), and as a proof of concept, we decided to start with the smallest pre-trained model and the vectors of 50 dimensions.

With this new version we are able to take advantage of resources from SemEval (12). SemEval is an international workshop in automatic semantic evaluation in which one of the tasks is to grade the semantic similarity between two sentences, giving them a similarity score. Using the SemEval corpus to test the semantic similarity engine can provide us a comparison of how good is our engine to discover semantic similarities in a general context. It is true that the Semeval corpus is based on very general sources and that is not directly comparable to the biomedical text, but the new proposed similarity engine relies not just on context-dependent metrics but in context-independent ones as well. For example, the syntactic metrics can be evenly applied in different literature contexts. Moreover, if we train a context specific word-embedding model, the overall semantic similarity measurement strategy it is not going to change, therefore it is very valuable to test it on a general task like Semeval task 1. Training the system with biomedical data would be a key aspect to consider and we must search for a strategy and the relevant corpus to evaluate the system in biomedical texts.

In the SemEval challenge, the maximum allowed score is five, meaning that both phrases convey exactly the same meaning, and the minimum score is zero, when the semantic of the phrases are completely independent. In the workshop the scores given by the systems are then compared against a gold standard manually assigned by human judges. Currently training data and test data, including gold standards, from year 2012 to 2015 are available, comprising around of 20 000 sentences and their human assigned score. We are preparing the new semantic comparator version in order to be easily configured to receive these sentences and give a result score in the SemEval scale. Then the system performance can be computed by calculating the Pearson correlation between the system results and the gold standards. This evaluation has the advantage of allowing us to compare the performance of our system against the performance of systems in previous years of SemEval and in this way give us a global picture of how good our semantic comparator engine is.

Clearly, we expect that performance will change when we consider biomedical texts. SemEval data sets are mainly formed by relatively short sentences extracted from different domains, whereas, sentences from biomedical articles could be larger and more complex. However, there is a lack of gold standards in biomedical domain to be used to compare the system.

## The ODIN interface

**Figure 3.**
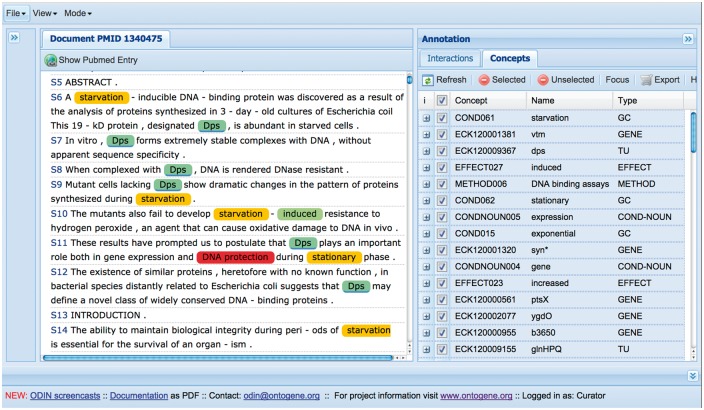
A screenshot of the curation system’s interface.

The results of the OntoGene text mining system are made accessible through a curation system called **ODIN** which allows a user to dynamically inspect the results of their text mining pipeline (see Figure 3). A previous version of ODIN was used for participation in the ‘interactive curation’ task (IAT) of the BioCreative III competition (1). This was an informal task without a quantitative evaluation of the participating systems. However, the curators who used the system commented positively on its usability for a practical curation task. An experiment in interactive curation has been performed in collaboration with curators of the PharmGKB database (8, 25). The results of this experiment are described in (20), which also provides further details on the architecture of the system.

**Figure 4.**
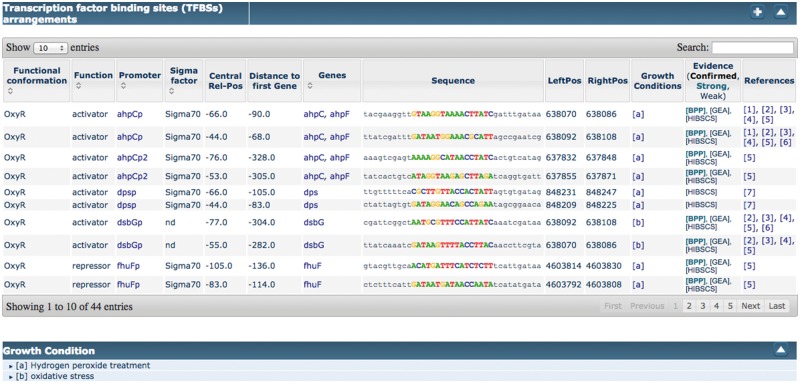
Example of novel growth conditions added to RegulonDB.

Another adaption of ODIN was performed for the Comparative Toxicogenomics Database (CTD) in the context of the BioCreative 2012 evaluation challenge (16). Although the goal of the challenge was to evaluate triage and information extraction capabilities of the participating text mining systems for the purpose of CTD curation, we also adapted our user interface, allowing curation of arbitrary PubMed abstracts using CTD entities and relationships.

At first access, the user will be prompted for a ‘curator identifier’, which can be any string. Once inside ODIN two panels are visible: on the left the article panel, on the right the results panel. The panel on the right has two tabs: concepts and interactions. In the *concept* tab a list of terms/concepts is presented. Selecting any of them will highlight the terms in the article. In the *interactions* panel the candidate interactions detected by the system are shown. Selecting any of them will highlight the evidence in the document. Selecting any concept or interaction in the results panel will highlight the supporting evidence in the article panel. Selecting any term in the article panel prompts the opening of a new panel on the right (annotation panel), where the type assigned to the term can be modified (or removed) if needed. It is also possible to add new terms by selecting any token or sequence of tokens in the article.

**Figure 5.**
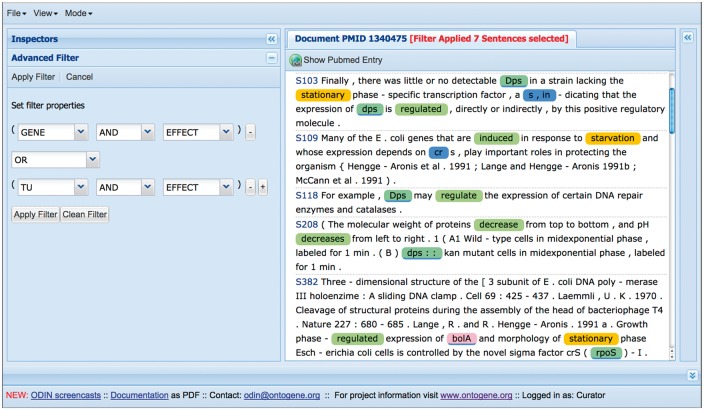
Using filters to focus on sections of the text more likely to contain the desired information.

Among the recent innovations for curation of RegulonDB, one of the most useful is the introduction of ‘filters’, which can be used by curators to restrict the amount of text that they have to inspect (see Figure 5). A filter in the sense defined by ODIN is simply a selector of sentences that satisfy a simple logical condition, such as containing at least one mention of a TF, and a mention of an effect, which is taken from our collection of words related to the type of regulatory effect of the TF on the regulated gene (e.g. ‘activates’, ‘activation’, ‘repress’, ‘repression’, ‘regulated’, ‘regulate’, ‘upregulates’). Filters can be easily defined by curators, using forms included within the ODIN interface, according to their needs. The usage of filters reduces the amount of text that a curator has to inspect in search for a particular item. The usefulness of filters was previously demonstrated through an experiment aimed at the identification of *‘growth conditions (GC)’* in which RIs are active (5). GC had not been curated before, and were therefore not part of the RegulonDB database. Compared with the version presented in the cited article, filters have now been extended to allow the definition of a more complex logical condition. Concretely, previously we had only the possibility to define a simple AND condition (the presence of two or more desired items in the same sentence). Now filters can include OR and NOT conditions, allowing a more flexible specification.

**Figure 6.**
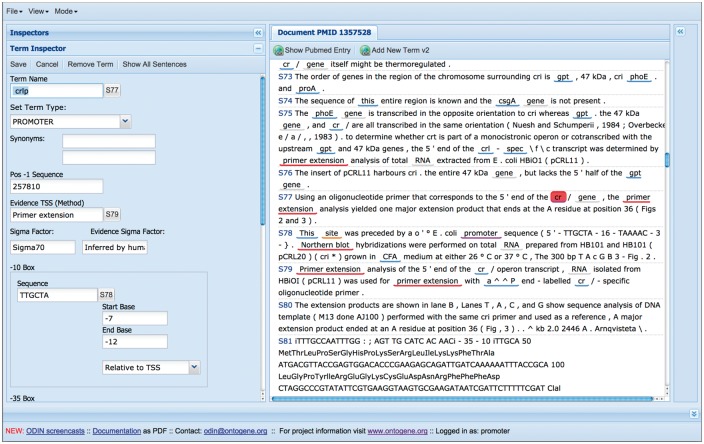
Self-filling forms can be used to speed up the curation process.

A more recent innovation is the introduction of partially self-filling forms for curation of specific types of entities (see Figure 6). When the curator identifies in text a novel entity that he wishes to curate, he can open a specific form by simply clicking on the corresponding text item, and picking one of the options for the entity type. Depending on the type of the entity, a pre-defined form will appear, and the system will try to fill the fields of the form using knowledge derived from the context in which the item appears and the background knowledge present in the RegulonDB database. The curator is then free to edit those fields in case any error is spotted, and once he is satisfied of the correctness of all the fields, can save the content of the form, which will be stored together with the document in a format which can later be used for exporting it to RegulonDB and EcoCyc. While a couple of such forms have already been implemented, we are in the process of designing them for all the objects of relevance in RegulonDB: gene, gene product, promoter, TF, TU, RI, GC, TF conformation, terminator, Effector, small RNA and ppGpp-DksA regulation. For each of the forms, a specific set of attributes is defined, for example for promoter, the following are considered: promoter name, synonyms, transcription start site (TSS), evidence of the TSS, promoter sequence, sigma factor, sigma factor evidence, -10 and -35 boxes sequence, TU name, comments, promoter evidence and references.

**Figure 7.**
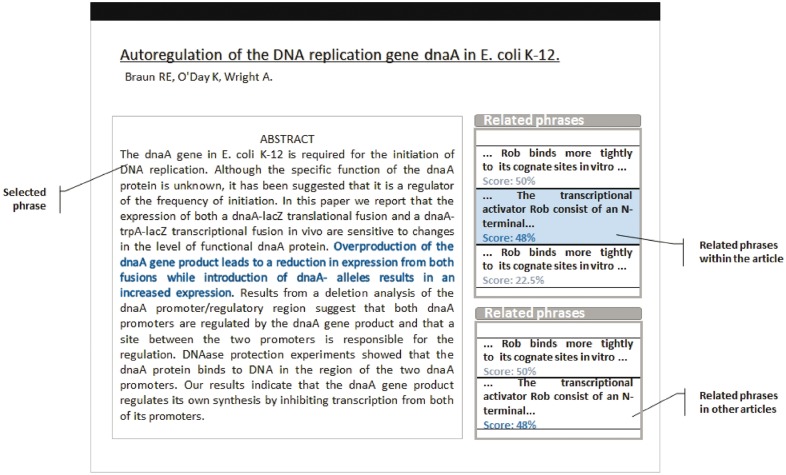
Preliminary version of the new interface, including cross-document semantic linking.

We are currently designing a novel web interface which will integrate ODIN functionalities with the semantic linking capabilities described at the end of Section 3 (see Figure 7). This new interface provides to the user the means to search key words on one or several articles. Once the results are presented, and the user decides to select one of the listed articles, the article’s sentences (content) are displayed, and those which have semantic relations with other sentences, either in the same article or in others, are decorated with hyperlinks. When the user selects a hyperlink, the related sentences that are located inside the current article are highlighted, and those which reside in others are listed in a panel along with the article name and the corpus to which it belongs to. In that way the user is provided with an instrument to navigate across different articles and corpus as a means to follow up on a specific idea.

The linking functionality is aimed to provide a way of navigating to other publications within the same corpus to which the publication belongs to. The semantic links are computed as an offline process that is triggered when the publication is integrated into the corpus, so the linking functionality would be already available when the user performs the curation.

## Results

This article describes primarily technical innovations in the curation process of RegulonDB. The improvements generated by these innovations feed into novel results which have been independently tested and published.

An initial version of the improved system was used in an experiment aimed at the identification of *‘GC’* in which RIs are active (5). An initial set of articles, likely to be relevant for the given task, was identified through conventional information retrieval (IR) techniques. The curators used ODIN filters to restrict their view of the selected articles to the set of sentences satisfying a given logical condition (e.g. containing an entity of type *‘transcription factor’* and an entity of type *‘effect’*). The manual analysis of the selected sentences allowed them to identify the missing information in 75% of the cases, but having to inspect only ∼10% of the articles, thus providing a considerable improvement in efficiency (5). Thanks to this strategy, GC for 53 RIs that involved three TFs (OxyR, SoxS and SoxR) were added to RegulonDB. More recently, a similar approach has been used to obtain GC for RIs concerning 28 TFs in RegulonDB. This experiment has once again shown a considerable improvement in curation efficiency compared with manual curation.

A separate experiment has been performed using the methods described in this article to curate microRNAs potentially involved in a pulmonary disease (idiopathic pulmonary fibrosis) (2). This experiment has shown an improvement of curation efficiency of up to 12 times. The unexperienced curators used for that experiment could not curate >1 article per day using the traditional methodology. Using the novel approach supported by the tools described in this article, it was possible in some cases to curate up to 12 articles per day.

Finally, the sentence similarity approach has been evaluated through an experiment aimed at finding if navigating across different articles guided by similar sentences could help the curators to efficiently identify specific information without having to read the whole documents. We tested the system using three sets of scientific articles:

1. 42 articles of SoxRS: oxidative stress in *E.coli* K12
2. 35 articles of *Salmonella typhimurium* pathogenicity island SP1
3. 10 articles of role of EZH2 gene in cancer

We had six domain experts that worked with these sets (two per set). The goal for the test exercise was to read the articles looking for specific information, as it is done in the curation process. Then, the experts had to extract and save all the information they could find within 2 h. One person from each set had access to the system, the other did not, instead they were provided with the PDF files. The users that had access to the system were able to review more articles, so they extracted in total more sentences with similar information. The users with the files could not read all articles within the given time, but they extracted more sentences per reviewed article.

The general opinion from the experts was that the system could be very powerful if the similarity is improved to detect more topic-related sentences. They also made some suggestions to the web interface in order to be more intuitive. The prototype has proved to be useful for the curation process, and we are now working to add more capabilities, improve the interface design by implementing User eXperience (UX) techniques, and integrate all components in a single unified system. We are also working on enhancing the similarity score and proposing a way to measure the quality of relationships.

## Conclusions

As part of our activities within the scope of the NIH-funded project ‘High-Throughput Literature Curation of Genetic Regulation in Bacterial Models’ we are implementing a process of digital assisted curation, which involves the integration of advanced text mining techniques within the curation pipeline of the RegulonDB database. Human experts will be able to leverage upon the best results of text mining technologies in order to improve the effectiveness of the curation process without sacrificing its quality.

The previously existing OntoGene text mining pipeline has been adapted and customized in order to provide entity recognition services for RegulonDB. The ODIN curation interface has been enriched with new functionalities in order to speed up the process of curation, in particular content filters that allow curators to quickly locate the information that they need, and partially self-filling forms which allow them to insert new entities and their metadata in the database. Additionally, we are in the process of integrating a method to interlink information across several articles. The new system will constitute a very powerful curation tool that will allow semiautomatic data annotation, and a new way of knowledge discovery, reducing reading time without affecting understanding.

## Funding

National Institute of General Medical Sciences of the National Institutes of Health (Award Number R01GM110597). The content is solely the responsibility of the authors and does not necessarily represent the official views of the National Institutes of Health. The OntoGene group at the University of Zurich is partially supported by the Swiss National Science Foundation (grant CR30I1_162758); F. Hoffmann-La Roche Ltd, Basel, Switzerland.

*Conflict of interest*: None declared.
